# Analysis of Candidate Colitis Genes in the *Gdac1* Locus of Mice Deficient in Glutathione Peroxidase-1 and -2

**DOI:** 10.1371/journal.pone.0044262

**Published:** 2012-09-06

**Authors:** R. Steven Esworthy, Byung-Wook Kim, Guillermo E. Rivas, Thomas L. Leto, James H. Doroshow, Fong-Fong Chu

**Affiliations:** 1 Department of Radiation Biology, Beckman Research Institute of City of Hope, Duarte, California, United States of America; 2 Molecular Medicine, Beckman Research Institute of City of Hope, Duarte, California, United States of America; 3 Laboratory of Host Defenses, National Institute of Allergy and Infectious Diseases, National Institutes of Health, Rockville, Maryland, United States of America; 4 National Cancer Institute, National Institutes of Health, Bethesda, Maryland, United States of America; Charité-University Medicine Berlin, Germany

## Abstract

**Background:**

Mice that are deficient for glutathione peroxidases 1 and 2 (GPX) show large variations in the penetrance and severity of colitis in C57BL/6J and 129S1/SvImJ backgrounds. We mapped a locus contributing to this difference to distal chromosome 2 (∼119–133 mbp) and named it *glutathione peroxidase-deficiency-associated colitis 1* (*Gdac1*). The aim of this study was to identify the best gene candidates within the *Gdac1* locus contributing to the murine colitis phenotype.

**Method/Principal Findings:**

We refined the boundaries of *Gdac1* to 118–125 mbp (95% confidence interval) by increasing sample size and marker density across the interval. The narrowed region contains 128 well-annotated protein coding genes but it excludes *Fermt1,* a human inflammatory bowel disease candidate that was within the original boundaries of *Gdac1*. The locus we identified may be the *Cdcs3* locus mapped by others studying *IL10*-knockout mice. Using *in silico* analysis of the 128 genes, based on published colon expression data, the relevance of pathways to colitis, gene mutations, presence of non-synonymous-single-nucleotide polymorphisms (nsSNPs) and whether the nsSNPs are predicted to have an impact on protein function or expression, we excluded 42 genes. Based on a similar analysis, twenty-five genes from the remaining 86 genes were analyzed for expression-quantitative-trait loci, and another 15 genes were excluded.

**Conclusion/Significance:**

Among the remaining 10 genes, we identified *Pla2g4f* and *Duox2* as the most likely colitis gene candidates, because GPX metabolizes PLA2G4F and DUOX2 products. *Pla2g4f* is a phospholipase A2 that has three potentially significant nsSNP variants and showed expression differences across mouse strains. PLA2G4F produces arachidonic acid, which is a substrate for lipoxygenases and, in turn, for GPXs. DUOX2 produces H_2_O_2_ and may control microbial populations. DUOX-1 and -2 control microbial populations in mammalian lung and in the gut of several insects and zebrafish. Dysbiosis is a phenotype that differentiates 129S1/SvImJ from C57BL/6J and may be due to strain differences in DUOX2 activity.

## Introduction

Mice deficient in the glutathione peroxidase isoenzymes, GPX1 and GPX2 (*Gpx1/2*-Double Knock Out [DKO]) have spontaneous ileocolitis that is driven by gut microbiota [1], [2]. Both proteins are members of the selenium-dependent GPX family and are classic antioxidant enzymes that reduce potentially noxious H_2_O_2_ and fatty acid hydroperoxides to water and alcohols. In humans, fourteen genes affecting oxidative stress, including *GPX1* and *GPX4*, are candidate genes for inflammatory bowel disease (IBD) and oxidative stress has been associated with IBD [3], [4]. Although the *GPX2* gene is hypomorphic [5], it is regulated by nuclear factor erythroid-derived 2-like 2 (NFE2L2/NRF2) [6]. An *NFE2L2*/*NRF2* gene promoter polymorphism is associated with ulcerative colitis in a Japanese population, implying that GPX2 may modify IBD [7]. Thus, *Gpx1/2*-DKO mice may represent an extreme case of oxidative stress-associated intestinal inflammation useful for understanding how oxidative stress affects IBD.

The impact of the *Gpx1/2*-DKO construct is dependent on mouse strain background. Colitis in C57BL/6 (B6) mice is rare, and it is mild when it occurs. In contrast, colitis occurs with 90% penetrance in the 129S1/SvlmJ (129) strain and often leads to morbidity before weaning. We reported that the *GPX-deficiency-associated-colitis 1* locus (*Gdac1*), containing the B6 allele, confers resistance to colitis in the 129 background. *Gdac1* maps to chromosome (Chr) 2: 119–133 mbp [8]. The large size of the reported interval accounts for differences in the 95% confidence intervals (CI), calculated by R/QTL, among the four phenotypes used to characterize strain difference in colitis susceptibility. The *Gdac1* locus overlaps *cytokine-deficiency-induced-colitis susceptibility 3* locus *(Cdcs3)*, which was localized based on mapping in resistant B6 *IL10*-KO vs. sensitive C3H/HeJBir (C3H) *IL10*-KO mice [9]. Here we compared nsSNPs in the genes at *Gdac1* and *Cdcs3* locus to determine the likelihood that *Gdac1* replicates *Cdcs3*.

The original *Gdac1* locus covers a region containing ∼300 genes, which makes thorough candidate analysis a daunting task. It contains two gene clusters separated by a gene-sparse region (122.6–124.4 mbp). The distal 125–133 mbp region includes a candidate human IBD gene, *FERMT1*, which encodes kindlin-1 localized at focal adhesions [10], [11], a spermine oxidase gene (*Smox*), an antioxidant transporter for ascorbate uptake (*Slc23a2*), several immunity genes (*IL1a*, *IL1b* and *Sirpa*), a major cell-cycle check-point gene (*Bub1*; marker SNP at 127.65 mbp) and a proliferative-cell-nuclear antigen gene (*Pcna*). Any of these genes could be envisioned to modify disease in *Gpx1/2*-DKO mice based on the colon pathology (crypt apoptosis, hyper-proliferation, acute inflammation, chronic inflammation, dysbiosis and/or antioxidant deficiency). The proximal region, ∼119–124 mbp, contains an equally compelling list of candidates, whose functions mirror those of the distal candidates. For example, BUB1B and CASC5 produced from the proximal region physically associate with BUB1 produced from the distal region to regulate mitosis [12]. The proximal region has two dual oxidase (*Duox*) genes, whereas the distal region has the *Smox* gene, all three oxidases generate H_2_O_2_ [13]. The proximal region has two potential autophagy genes, encoding vacuolar protein sorting (VPS)-18 and -39, whereas the distal region encodes VPS16 [14].

Here we report our analysis of 155 additional mice with an increased number of single-nucleotide polymorphism (SNP) markers throughout the *Gdac1* region. The analysis eliminated the distal 126–133 mbp of the original locus from consideration, essentially halving the number of potential genes. The common ancestry of the proximal region in C3H and 129 better supports the notion that *Cdcs3* replicates the proximal region of *Gdac1* rather than the distal region. Using *in silico* analysis, we evaluated 128 well-annotated protein-encoding genes, largely from the proximal region as well as 1 putative and 2 validated microRNA (miRNA) genes. Based on gene function in the colon and/or pathology observed in DKO mice, we selected the top 25 protein-encoding colitis gene candidates for e-QTL analysis. In this report, we summarize the process used for gene selection and elimination. We then explain the rationale for why we chose *Pla2g4f* and *Duox2* as the top colitis gene candidates rather than eight other genes that included *Bub1b*, *Plcb2*, *Casc5*, *Chac1*, *Oip5, Pla2g4e*, *Trp53bp1* and *Slc28a2*.

## Results

### Refined Mapping of the *Gdac1* Locus

**Figure 1:**
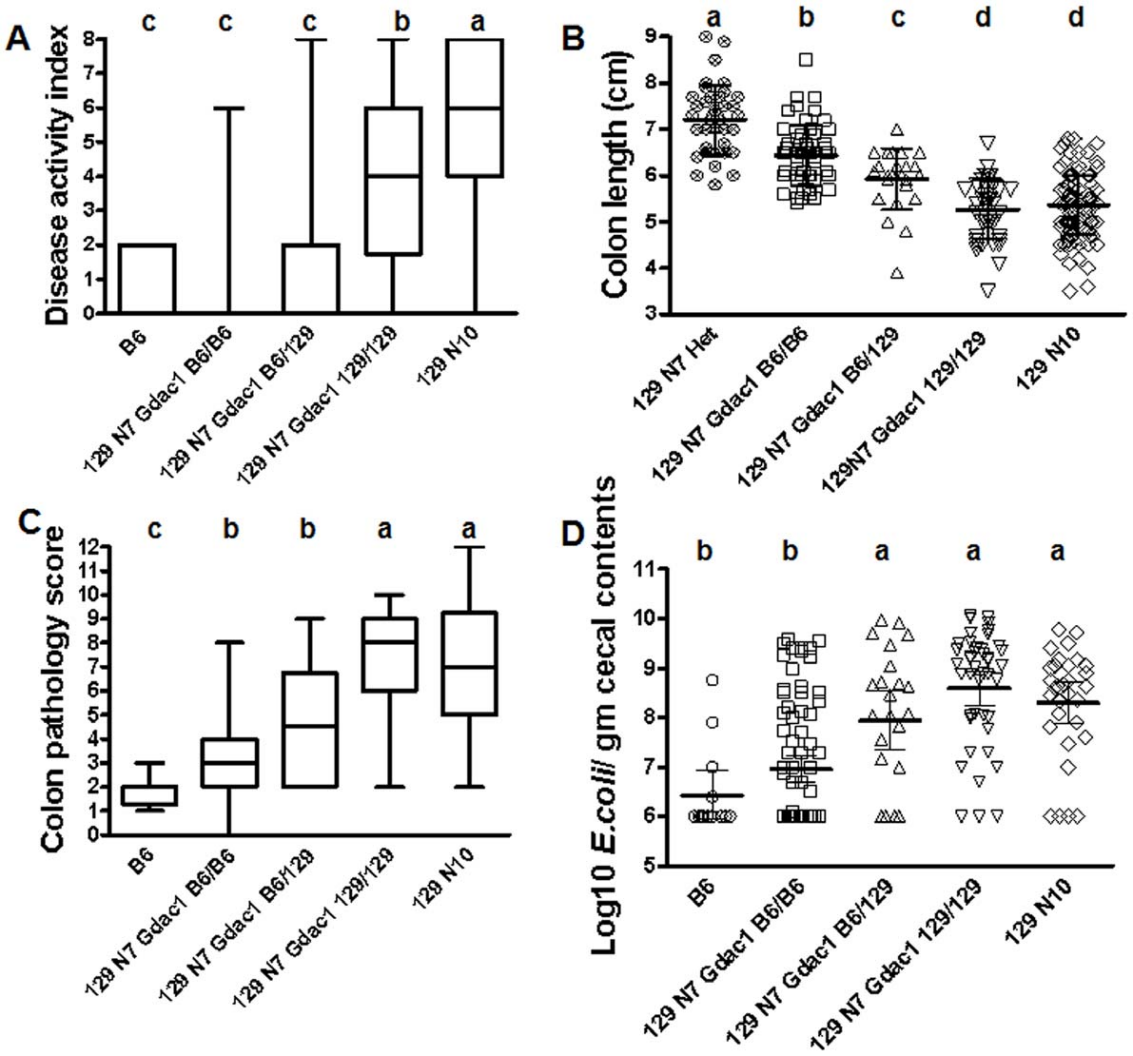
Phenotypes used to refine the *Gdac1* locus. Panel A. A box-and-whisker plot of disease activity index of DKO mice grouped by strain background and *Gdac1* genotype. *Gdac1* alleles were based on typing at *Rpusd2* (118.8 mbp) and *Duox2* (122.1 mbp) and/or *Slc30a4* (122.5 mbp). Statistics was evaluated with 1-way ANOVA (Kruskal-Wallis); Dunn’s post test; α = 0.05. Different letters above the boxes indicate means are different, where a>b>c. The number of mice in each group is 16, 74, 35, 38 and 233, from left to right. Panel B. A scatter plot of colon lengths. B6 DKO mice were not included as a reference because they and the B6 WT colon lengths fell in the middle of the collective129 data. This might be contributed by the smaller stature of B6 mice at weaning. Instead, *Gpx1−/−Gpx2+/−* littermates of the *Gdac1* N7 DKO mice were used as the reference (129 N7 Het). One-way ANOVA; Tukey’s post test; N = 39, 56, 23, 43, 119. Different letters above the boxes indicate means are different, where a>b>c>d. Panel C. A box-and-whisker plot of colon pathology scores. One way ANOVA (Kruskal-Wallis), Dunn’s post test; N = 21, 52, 24, 38, 54. Different letters above the boxes indicate means are different, where a>b>c. Panel D. A scatter plot of Log10 CFU *E. coli* per gm cecal contents. One-way ANOVA (Kruskal-Wallis), Dunn’s post test; N = 14, 79, 24, 42, 31. Different letters above the boxes indicate means are different, where a>b.

**Figure 2:**
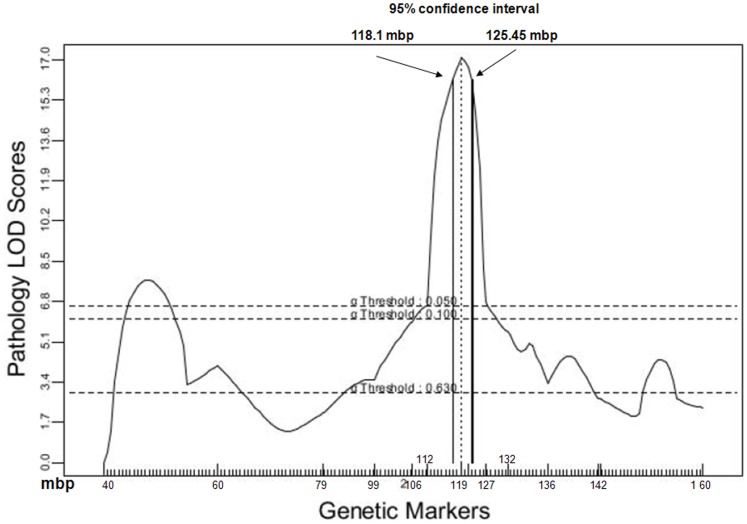
LOD plot for the distal colon pathology score phenotype across Chr 2 from 40.5 to 160 mbp. Marker locations (mbp from centromere) are indicated on the X axis. Arrows demark the 95% CI. LOD plots for colon length, DAI and Log10 *E. coli* CFU/gm cecal contents are in Figure S1, S2, S3.

The increased marker SNP density enabled us to detect multiple recombination events between the original markers at 118.8 mbp and 142 mbp and estimate the location of recombination within the *Gdac1* interval (Table 1). The increased numbers of mice provided more recombinants in the interval for analysis. The impact was that the 95% confidence interval (CI) was limited to the region of 118 mbp to 125 mbp with good agreement among the four phenotypes. The phenotypes measured were disease activity index (DAI; the criteria are defined in Table 2), colon length, colon pathology score (H&E histology) and *E. coli* overgrowth (Fig. 1). R/QTL calculated LOD ranged from ∼11–21 within the 95% CI (Fig. 2 and Fig. S1, S2, S3).

**Table 1:** Additional SNP markers for this study.

Location	Reference SNP	Gene	Comment
Chr1: 87687800	rs32549111	–*	polymorphism eliminated**
Chr1: 87838162	rs30671689	*Gpr55*	polymorphism eliminated
Chr1:1422002036	rs13459053	*Cfh*	polymorphism eliminated
Chr2: 40553058	rs27175338	*Lrp1b*	no polymorphism expected
Chr2: 79162839	rs28305948	*Itga4*	
Chr2: 106189893	rs3148954	*Dcdc5*	
Chr2: 112319953	rs27491511	*Chrm5*	
Chr2: 122117242	rs27453362	*Duox2*	
Chr2: 122509506	rs2650268	*Slc30a4*	backup for *Duox2*
Chr2: 127649702	rs2826904	*Bub1*	
Chr2: 132737018	rs2724570	*Fermt1*	
Chr2: 136711224	rs2726691	*Mkks*	
Chr3: 11824234	rs29713670	–	no polymorphism#
Chr8: 64173319	rs33548981	*Palld*	no polymorphism

* “-” means there is no gene with the SNP. Analysis in R/QTL showed no association of chromosome 1 alleles with disease phenotypes.

** Selective incrosses were set up to eliminate B6 alleles at these loci.

# B6 alleles indicated by genome-wide analysis are not detected by in-house markers and methodology.

**Table 2:** Disease activity index.

Score*	Growth or weight loss	Diarrhea index
0	On par with non-DKO littermates	None
1	Growth arrest**	
2	10% loss	Wet tail#
3	10% to 20% loss	
4	≥20% loss¶	Diarrhea§

* The final score is based on adding the results from the Growth and Diarrhea columns.

** Cessation of growth commencing on day 8 to 13.

# Matted and discolored fur at tail base. No accumulation of feces.

¶ Lethargy was not scored. It was taken into consideration to euthanize mice and occurred independent of scores.

§ Large accumulation of partially or completely dried stool at tail base.

The *Gdac1* gene count was cut by nearly one-half compared with our previous report [8]. Because many strong candidates were eliminated, we manually analyzed 4 genotypes of *Gpx1/2*-DKO mice to assess the results of the R/QTL analysis (Fig. S4A). We identified a group of mice that were 129/129 for markers at 118.8 and 122.1–122.5 mbp and B6/B6 at 127.6 and 132.7 mbp (the reciprocal was not found). This group shared the phenotypic properties of 129N10 mice (129/129 throughout the interval) and distinguished them both from N7 mice B6/B6 throughout the 118.8–132.7 mbp interval and a congenic line (B6/B6; 118.8–137 mbp) (*P*≤0.05; 1-way ANOVA; Fig. S4B–S4E). This was consistent with the R/QTL output, which indicated there was little impact on disease severity caused by any variation in the distal region. We have a congenic line in which the proximal end of the differential segment is delineated by a SNP marker at 118.8 mbp. We observed the congenic DKO mice were significantly healthier than the reference 129 N10 population based on all 4 phenotypes, which was consistent with the R/QTL analysis indicating that the proximal boundary of *Gdac1* is near 118 mbp.

### Influence of *Gdac1* on Dysbiosis

In a previous study, we examined the composition of the microflora in the ceca of WT and *Gpx1/2*-DKO mice of the B6 and 129 strains [15]. This was determined by non-culture-based, automated ribosomal-intragenic-spacer analysis on DNA isolated from total cecal contents. The consistent feature was the overgrowth of *E. coli* and *Enterococcus sp.* in the 129 *Gpx*1/2-DKO mice that suggested their association with pathology. *E. coli* overgrowth had high enough penetrance in 129 DKO mice to be a good marker for dysbiosis, whereas *Enterococcus* overgrowth had too low penetrance to be useful (Fig. 1D).

**Figure 3:**
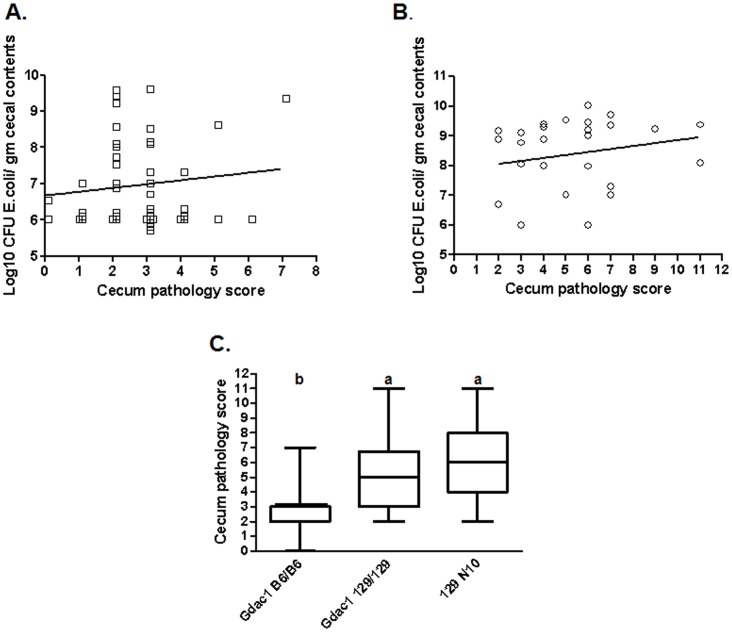
Correlation between *E. coli* overgrowth and cecum pathology in 129 N7 *Gpx1*/2-DKO *Gdac1* ^B6/B6^ mice (Panel A; N = 53) and *Gdac1* ^129/129^ mice (Panel B; N = 25). The slopes of the linear regression lines are not significantly different from 0. Data clustered at 6 for Log10 CFU have been offset on both the X and Y axes to show the points. Panel C shows the cecum pathology scores from Panel A and B in box-and-whisker format along with reference 129 N10 (N = 29). Different letters above the groups indicate significant differences (a>b; P<0.05; Kruskal-Wallis with Dunn’s multiple comparison test).

We found that *Gdac1* could influence *E. coli* overgrowth in the cecum (Fig. 1D, 3A and 3B). The cecum was a disease site, although it had milder disease than the distal colon (Fig. 1C and 3C). When we plotted bacterial CFU against pathology scores, we did not find any correlation between them within *Gdac1* genotype groups (*Gdac1^B6/B6^*, R^2^ = 0.013; *Gdac1^129/129^*, R^2^ = 0.04) (Fig. 3A and 3B). Therefore, dysbiosis was not a result of gross inflammation (pathology scores of 7 and above) but was associated with the underlying conditions that promoted apoptosis, hyper-proliferation and mucin depletion (all of which contributed to scores of 1 to 6). This was the first indication that *Gdac1* affected processes that precede or promote development of inflammation rather than affecting the intensity of inflammation.

### The Genetic Landscape of *Gdac1* and Relationship to *Cdcs3* and *Dssc2*

**Figure 4:**
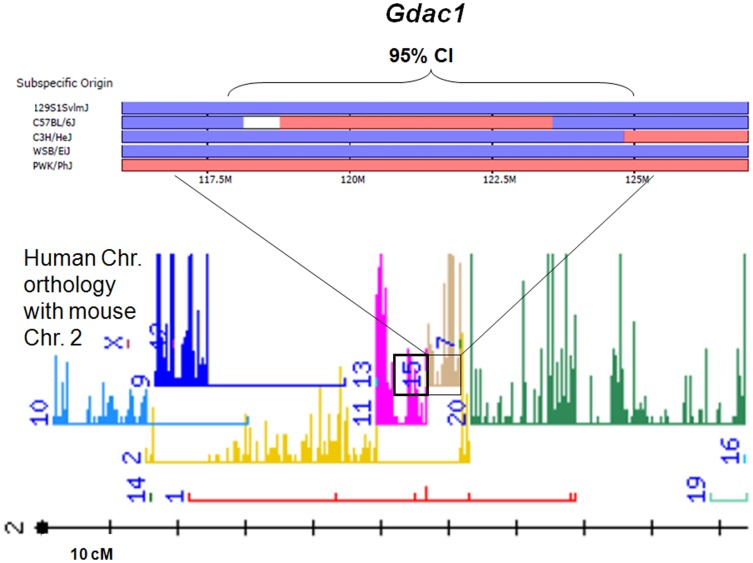
The subspecific origin of *Gdac1* and the relationship to a human locus. The upper portion of the figure shows chromosome ancestry analysis. The *Gdac1* 95% CI in B6 is derived from *M.m. musculus* (small section of indeterminate ancestry at proximal end; reference wild-derived *M.m. musculus* strain is PWK/PhJ). This contrasts with both 129 and C3H, where *M.m. domesticus* is the source of the chromosome (wild-derived WSB/EiJ is the reference *M.m. domesticus* strain). The boundaries of *Gdac1* coincide with a portion of human chromosome 15q (brown), shown in the lower portion of the figure (data retrieved from CGD- http://msub.csbio.unc.edu/ −and MGD at the MGI website; 04/2012-mouse phylogeny viewer and mouse; human orthology map).

*Gdac1* lies adjacent to *Cdcs3* and *Dssc2*, which are colitis loci that were identified and analyzed in B6 vs. C3H *IL10*-KO mice and dextran sodium sulfate (DSS)-treated wild-type (WT) mice, respectively [9], [16]. Our refined *Gdac1* was close to *Cdcs3* (peak LOD at 117.95 mbp; *Thbs1*; Table 3) and could be confidently distinguished from *Dssc2* (∼80 mbp). We found *Gdac1* was conserved in humans, who had a nearly identical gene list for Chr.15q: 38–49 mbp (Fig. 4).

**Table 3:** *Gdac1* QTL candidate genes (shortlist in proximal to distal order).

Gene	Location	Primers^∧^	nsSNPs**	PolyPhen-2**	eQTL#evidence	KO/mutant¶
*Spred1*	116947186	GAGATGACTCAAGTGGTGGATGTCTGAAAGGTAAGGCCAAACTTC	No		No	KO-airway eosinophilia
*Thbs1*	117937658	GCCGGCGGTAGACTAGGCCT GCGAGGAAAGGCGATCCCGG	Possible	All benign	No	KO-bad DSS response
*Bmf*	118354493	GGAGCGGGCGTATTTTGGAAACACTCGATTGGGAAGAAGGG	No		No	KO-urogenital defect
*Bub1b* *	118423992	GAGGCGAGTGAAGCCATGTTCCAGAGTAAAAGCGGATTTCAG	Yes		No	KO-hemopoetic defects
*Plcb2* *	118533253	AGGATAGCTGTGATGGAAGAAGGGCCCAGGTGTCAGGTATGTAG	Yes		No	KO-enhanced chemotaxis
*Bahd1*	118727351	GGGCAGCTTCTACCTGTACTGGCTGTAGCCATTCGCTCCA	No		No	KO- listeriosis decreased
*Rpusd2*	118860526	AGGACGCCTGCATCTCAACCGGCTTGTCTATAACAACCACAT	No		No	None
*Casc5* *	118872855	TCCGACTCCAAGGAGCGCGATGCAAAGCTGACTCGACGAGAGC	Yes		No	None
*Rad51*	118938553	GTCCACAGCCTATTTCACGGTACAGCCTCCACTGTATGGTAAC	No		No	KO-embryonic lethal
*Spint1*	119063096	CCAGTGTGCTCCGGCAGCTGGTGGCCTCGTCGGAGCCATC	No		No	KO-peri-natal lethal
*Vps18*	119124189	ATACACTGCTCCGCATTGACTGGTTCATGTAAAGGACCTCGGT	No		No	(Zebra Fish)
*Dll4*	119151565	CAGTTGCCCTTCAATTTCACCTAGCCTTGGATGATGATTTGGC	Yes	All benign	No	KO-no impact (complements Dll1)
*Chac1* *	119176992	CTGTGGATTTTCGGGTACGGCTCGGCCAGGCATCTTGTC	Possible		No	None
*Ino80*	119198778	CCACACAACAAGCACAGAACAGAGACCACGTTTCTTGCCG	No		No	None
*Oip5* *	119435268	CTCGTCTAAACTCACTGACGGGCAGGATCTTTGGTGATGCTGTT	Yes		No	None
*Mapkbp1*	119798435	AGAAGGGTCAACCATTACTAGCCGCTGGGTAGGCAACTAAGCC	Yes	All benign	No	None
*Pla2g4b*	119860328	CGGCCAGCCTCCAAGACAGCAGAGCTCGGCGATCCTGCCA	Yes	All benign	No	None
*Pla2g4e* *	119992148	AACGGCGTGCTGGTGTCTCGAGGCGCCTCTGCGTAAAGCTGAAACCCATGTGAAGTTCCCACCCTCCAGGCAAGAGACTTGG	Yes		Yes	None
*Pla2g4f* *	120125702	CCCAGTGCTGAGCCCCAAGCTGCTGGCCCTCCTCCAGGTC	Yes		No	None
*Trp53bp1* *	121023987	ATTGAACGGTTACCTCAGCCACCCAACTGTGATGAAGCAGAAT	Possible		No	KO-immune deficient
*Duox2* *	122106173	TCACAACGGACGGCTTGCCCCCCGGCCACTCCATTGCTGG	Yes		No	Mutant-hypothyroid
*Duoxa2*	122124636	GCACTCGCGCTGGTTCTGGTATGTTAACGCCCGCCAGCCC	No		No	Mutant-hypothyroid
*Slc28a2* *	122252213	AGTGGAGAATTGCATGGAGAACGACCAAGCAGGATCTTTCTGAA	Possible		No	None
*Slc30a4*	122506975	GCTGACCATCGCTGCCGTCCTGGCCGACAAAACCTCTAGGCG	Yes	All benign	No	Mutant-lethal milk
*Cops2*	125656040	ATGAGGAGGACTACGACCTGGTGAATCCCCATTCTCCCTTCTC	No		No	KO-embryonic lethal

? The primers are listed at 5′ to 3′ direction. Each set of primers are listed with forward primer on the top and reverse primer in the bottom. Two primer sets were tested for *Pla2g4e*.

* means those genes are viable candidates.

** nsSNPs- “No” means no nsSNPs reported in databases for B6 vs. 129 and often among all strains. “Possible” refers to incomplete annotations for B6 and/or 129; an nsSNP was noted at this location involving at least one strain among all inbred strains in databases. For *Trp53bp1*, PolyPhen-2 rejected several amino acid calls, which prohibited SNP evaluation. The latter also applied to several other genes shown in Table S1. “All benign” means that all nsSNPs were predicted as “benign” by PolyPhen-2.

# Gene expression levels in colon examined by qPCR are normalized against β-actin or 36B4. P-values between 0.05 and 0.1 were rated as “Possible.” However the pattern of expression in the sets of mice often suggests that this was a reaction to pathology rather than true cis-e-QTL status.

¶ Knockout or mutants available for reference. “None”-no KO or mutant studies in mice, except *Vps18*-KO study was performed in Zebra fish. However, Online Mendelian Inheritance in Man (OMIM) reports at least 20 mutations/deletions in humans for this region (Chr: 15q 38–49 mbp) with associated syndromes.

The mouse phylogeny viewer (http://msub.csbio.unc.edu/) showed that the refined B6 allele of *Gdac1* coincided with a chromosome block that originated from *M.m. musculus* (PWK/PhJ), flanked by blocks that originated from *M.m. domesticus* (WSB/EiJ). For C3H and 129, which were used to define *Cdcs3* and *Gdac1* vs. B6, respectively, the entire stretch originated from *domesticus*. This suggested that *Gdac1* and *Cdcs3* could be replicates (Fig. 4). In fact, C3H and 129 shared haplotypes in the gene-dense 118.8–119.3 mbp region, which indicated sequence identity (Fig. S5). A similar circumstance arose at 123–124 mbp; this region had only 2 poorly annotated genes. Distal to 124 mbp, B6 shared ancestry with the129 and C3H strains. At ∼125–129 mbp, C3H did not share the common ancestry of B6 and 129. Thus, it appeared unlikely that *Cdcs3* and *Gdac1* had the same polymorphism in the region distal to 124 mbp.

### Preliminary Analysis of *Gdac1* to Identify Unlikely Candidate Genes

**Figure 5:**
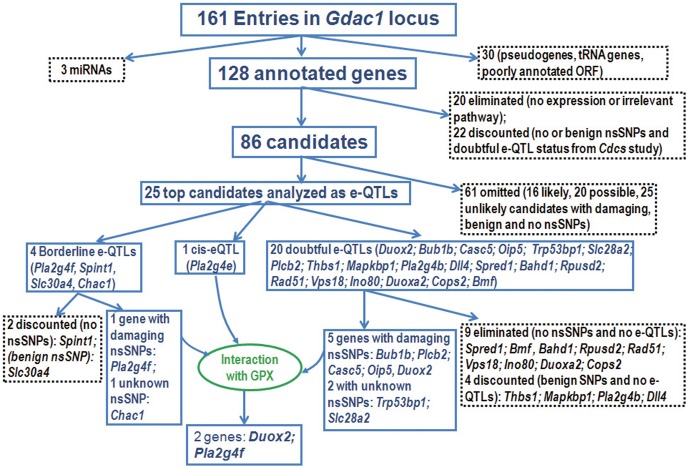
Outline of the candidate categorization process. The eliminated and discounted genes as well as the genes for 3 microRNAs and omitted genes are shown in dotted-black boxes. The viable candidates at each step are shown in solid blue boxes.

There were 128 well-annotated, protein-encoding gene entries in the *Gdac1* region (161 total entries before we excluded pseudogenes, tRNA genes, miRNA genes and poorly annotated, presumptive open-reading frames) (Fig. 5 and Table S1). Non-coding RNAs are discussed in a separate section, below. Sixteen genes were eliminated by *in silico* analysis based on lack of expression in the colon or involvement in pathways that fall outside of those implicated in IBD (flagged as N in column 14 and “pathway” or “no expression” in column 15 and highlighted in grey in Table S1) [3]. Below are examples of what we considered to be irrelevant pathways. A *Tyro3*_47_ knockout affected spermatogenesis by disruption of Sertoli cell-specific signaling pathways (the subscript 47 refers to the gene number in Table S1) [17]. *Cdan1*_66_ defects produced anemia without other discernable effects [18]. The codanin-1 protein chaperones the heterochromatin protein 1 homolog α from the Golgi to the nucleus in erythroblasts [19], and *Myef2*_118_ codes for a factor that regulates the myelin basic protein gene [20]. An additional 4 genes were eliminated based on inference from gene deletions or mutations in human studies. *Fbn1*_123_ codes for fibrillin-1; deletions in this gene are implicated in aneurysm [21], [22]. *Tmg5*_72_, *Spg11*_101_ and *Cep152*_124_ defects likewise produced human syndromes (skin peeling, spastic paraplegia and Seckel syndrome, respectively) without impacting the gastrointestinal (GI) tract [23], [24], [25]. Seven of the 20 genes (*Tyro3*_47_, *Cdan1*_66_, *Strc*_82_, *Catsper2*_83_, *Spg11*_101_, *Slc24a5*_117_ and *Slc12a1*_120_) were eliminated using multiple criteria that included analysis of human gene mutations, results obtained from mouse or zebrafish knockouts, involvement in irrelevant pathways or lack of expression in the colon [17], [18], [24], [26], [27], [28], [29], [30], [31].

Thirteen genes (*Rasgrp1*_3_, *Srp14*_9_, *Ivd*_19_, *Snap23*_62_, *Lrrc57*_63_, *Ccndbp1*_70_, *Tubgcp4*_77_, *Ckmt1*_81_, *Pdia3*_84_, *Serf2*_86_, *Shf*_107,_ *Dut*_121_ and *Eid*_126_) were discounted based on lack of nsSNPs and doubtful status as e-QTLs (*Cdcs3* analysis by de Buhr *et al.*) [32]. Another 9 (*Eif2ak4*_8_, *Dnajc17*_28_, *Zfyve19*_30_, *Ndufaf1*_42_, *Stard9*_65_, *Ttbk2*_67_, *Ubr1*_68_, *B2m*_98_ and *Sqrdl*_114_) were provisionally discounted because PolyPhen-2 analysis indicated the nsSNPs were benign (i.e. did not affect protein function) and they had doubtful e-QTL status. PolyPhen-2 is a second generation web-based tool that evaluates the impact of amino acid substitutions caused by nsSNPs on protein function [33]. Because PolyPhen-2 analysis is not regarded as definitive (72% accuracy) and because we did not perform our own e-QTL analysis, these 22 genes cannot be eliminated from candidacy [34]. In order to eliminate a discounted gene, we would need to demonstrate that it 1) lacks nsSNPs and has no e-QTL status; or 2) has benign nsSNPs and has no e-QTL status. In summary, we eliminated 20 genes and discounted 22 from candidacy.

### Selection Criteria for e-QTL Analysis of *Gdac1* Candidates

Based on gene function in the colon and/or pathology observed in DKO mice, we selected 25 genes from the remaining 86 candidates for e-QTL analysis (Table 3). Among the 86 genes, there were at least 11 genes in the DNA repair and mitosis spindle formation/regulation/check-point group (*Bub1b*_11_, *Casc5/*Blinkin_24_, *Rad51*_25_, *Ino80*_37_, *Oip5/*Lint-25_40_, *Nusap1*_41_, *Trp53bp1*_78_, *Cep152*_124_, *Haus2/*Cep27_64_, *Ccndbp1*_70_ *and Tubgcp4*_77_ *)* [35]. We selected six; *Bub1b*_11_, *Casc5*_24_, *Rad51*_25_, *Trp53bp1*_78_, *Ino80*_37_ and *Oip5*_40_. *Cep152*_124_ was eliminated because in humans a mutation causes Seckel syndrome without specific GI symptoms [25]. *Nusap1*_41_, *Tubgcp4/D2Ertd435e*_77_ *and Ccndbp1*_70_ were analyzed by de Buhr *et al.* as e-QTLs and did not reach the 1.5x threshold of biological significance [32]. *Haus2*/Cep27_64_ was not analyzed in order to allow analysis of genes in other pathways.

The H_2_O_2_-producing oxidases DUOX1 and DUOX2 are encoded by the *Duox1*_106_ and *Duox2*_103_ genes. They are clustered in *Gdac1* with their respective maturation factors, *Duoxa1*_105_ and *Duoxa2*_104_ [36]. *Duox1* is expressed at a very low level in the intestine, while *Duox2* is readily detectable ([37] and data not shown). There is a cluster of three *Pla2g4* genes producing intracellular PLA2s, and a PLA1, which is encoded by *Pla2g4e*_54_ [38]. *Pla2g4d*_55_ is not expressed in the intestine, and is involved in psoriasis [39], [40], [41]. Therefore, we included *Duox2*_103_, *Duoxa2*_104_, *Pla2g4b*_51_, *Pla2g4e*_54_ and *Pla2g4f*_56_ in further analysis (Table 3).

Seven genes, *Spred1*_1_, *Thbs1*_4_, *Bmf*_10_, *Plcb2*_14_, *Bahd1*_20_, *Mapkbp1*_50_ and *Slc28a2*_108_, were selected from 12 genes potentially linked to colitis through either immune signaling or suggestive evidence from DSS and/or gene knock out studies. The remaining five, *Rasgrp1*_3_, *Ndufaf1*_42_, *Ltk*_45_, *Pdia3*_84_ and *B2m*_98_, were previously analyzed as *Cdcs3* candidate e-QTLs but showed no differences in levels at the significance threshold of 1.5x [32], and so were not analyzed in this study.

Seven additional genes were selected based on either possible overlap with human IBD candidates (*Rpusd2*_23_), pathway defects implicated in IBD (*Vps18*_34_; *Chac1*_36_; *Cops2*_128_), involvement with epithelial differentiation and development (*Dll4*_35_) or involvement in mouse development (*Spint1*_32_; *Slc30a4*_112_). *Rpusd2* is a possible analog of a human IBD candidate, *PUS10*; *Spint1* regulates development; *Vps18* is in the autophagy pathway; *Dll4* encodes a Notch1 ligand; the *Chac1* product links ER stress to apoptosis; a *Slc30a4*_112_ mutant causes lethal milk (zinc deficiency); the *Cops2* protein is involved in the ubiquitin-proteasome COP9 signalosome that functions during general embryonic proliferation, T-cell development and T-cell antigen-stimulated proliferation [14], [42], [43], [44], [45], [46], [47].

### *Gdac1* Candidates Evaluated as e-QTLs

**Figure 6:**
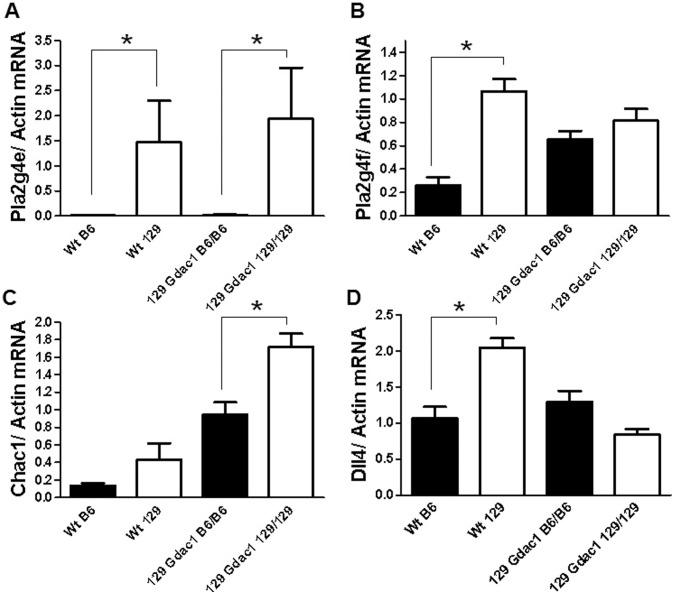
e-QTL analysis by RT-PCR. Relative mRNA levels of *Pla2g4e* (A), *Pla2g4f* (B), *Chac1* (C) and *Dll4* (D) in the distal colons of 129 strain *Gpx*1/2-DKO *Gdac1* mice by genotype and in WT B6 and WT 129. *Dll4* represents a common pattern among candidates, where the pattern suggests expression was affected due to pathology in the *Gdac1^129/129^* mice. For all panels, N = 6, 6, 6, 6. An * indicates significant difference, α = 0.05, for pair-wise t-tests between parental strains or between the *Gdac1* sets (B–D). *Pla2g4e* uses the non-parametric Mann-Whitney test, because the distributions were not Gaussian.

Among the 25 genes analyzed, *Pla2g4e*_54_ was the lone definitive cis-e-QTL found in this survey; RT-qPCR with two primer sets confirmed it had different expression levels between 129-*Gdac1^B6/B6^* and 129-*Gdac1^129/129^* DKO mice and between the parental strains (Fig. 6A). We and others found that B6 colon barely expressed *Pla2g4e* mRNA [41]. In contrast, the current results showed that the 129 colon does express the *Pla2g4e* mRNA. *Pla2g4f*_56_ was a borderline e-QTL (Fig. 6B); between B6 and 129 the difference in expression levels was significant, but between 129-*Gdac1^B6/B6^* and 129-*Gdac1^129/129^* mice the difference was not significant. However, the 129 allele had higher expression levels in replicate RT-PCR analyses using separate standards. *Pla2g4f* mRNA in B6 colon was detectable on Northern blots, a result consistent with the RT-PCR results [41]. The e-QTL status of *Spint1*_32_ and *Slc30a4*_112_ was identical to *Pla2g4f*. Because *Pla2g4e* and *Pla2g4f* also had predicted damaging nsSNPs, they were considered to be good candidates. *Spint1* had no nsSNPs and *Slc30a4* nsSNPs were rated as benign, so their candidacy was discounted. *Chac1*_36_ (Fig. 6C) was a fourth borderline e-QTL. The 3-fold difference in gene expression levels between the parental strains was not significant. The difference between the *Gdac1* DKO mice sets was significant and consistent with the trend observed in the parental strains. The borderline e-QTL status and uncertainty about the *Chac1* nsSNPs resulted in the gene being classified as undecided. However, the fact that *Chac1* responds to oxidative stress indicated that it may warrant further analysis. Another 7 genes (*Bub1b*_11_, *Plcb2*_14,_ *Casc5*_24_, *Oip5*_40_, *Trp53bp1*_78_, *Duox2*_103_ and *Slc28a2*_108_) were not e-QTLs, but they remained candidates because they had potentially damaging nsSNPs.

Due to negative or conflicting outcomes from our e-QTL analysis, we considered the potential candidacy of another 13 genes to be poor. In the case of the *Dll4*_35_ gene (Fig. 6D), the significant difference in levels between 129 WT and 129-*Gdac1^129/129^* mice (P<0.05) can be attributed to the loss of goblet cells, where it is expressed [48]. *Thbs1*_4_, *Dll4*_35,_ *Mapkbp1*_50_ and *Pla2g4b*_51_ were discounted due to nsSNPs being rated as benign, and/or no expression differences, or inconsistent expression differences. Nine genes (*Spred1*_1_, *Bmf_10_*, *Bahd1*_20_, *Rpusd2*_23_, *Rad51*_25_, *Vps18*_34_, *Ino80*_37_, *Duoxa2*_104_ and *Cops2*_128_ *)* were eliminated because they had no nsSNPs and showed either no expression differences or inconsistent expression differences.

Of the 25 genes selected for evaluation as e-QTLs, 10 remained as candidates (*Bub1b*_11_, *Plcb2*_14_, *Casc5*_24_, *Chac1*_36_, *Oip5*_40_, *Pla2g4e*_54_, *Pla2g4f*_56_, *Trp53bp1*_78_, *Duox2*_103_ and *Slc28a2*_108_). However, this was generally due to the nsSNPs found in these genes rated as damaging by PolyPhen-2. The clear exception was *Pla2g4e*; *Pla2g4f* and *Chac1* represented borderline e-QTLs. Among the 10, *Pla2g4f* and *Duox2* were prioritized as the strongest candidates because they were associated with oxidative stress through possible interactions with GPXs.

### Non-coding RNAs

There were 2 validated miRNAs in the MGI databases that mapped within *Gdac1*; *Mir674_1a_* (117 mbp) and *Mir147_111a_* (122.4 mbp). There appears to be one SNP within *Mir674* and 13 more were found within 2 kb of *Mir674* using available mouse strains. However, the B6 sequence is not yet determined and a function for *Mir674* has not been reported. There were no SNPs in *Mir147* but 4 were within 2 kb of its location and it is possible that 129 and B6 could have different variants at all 4 sites. *Mir147* is expressed in the spleen but not in normal colon and is involved with TLR4-stimulated macrophage production of TNFα and IL6 [49]. This possible anti-inflammatory role made *Mir147* a candidate colitis gene in *Gdac1* [50]. However the paucity of SNPs around the miRNA and within the AA467197/*NMES1* gene (7 SNPs within 2 kb), where *Mir147* resided, suggested that there may be no expression difference between 129 and B6.

In the corresponding human 15q region we identified 5 miRNAs (*miR-626*, *miR-4310*, *miR-627*, *miR-1282*, *miR-147B*) and one antisense RNA, *OIP5-AS1*. *miR-147B* is the human ortholog of *Mir147*. The regulation *of miR-147B* is distinct from *Mir147* suggesting a different function in humans [50]. We found a match for *miR-1282_87a_* in the *Gdac1* locus using BLAST (100% match; 100% coverage; ∼121.28 mbp). No matches were found for *miR-626*, *miR-4310, miR-627* and *OIP5-AS1* even with a lower stringency search. Collectively, there were two validated and one potential miRNA in *Gdac1*.

## Discussion

Based on the low LOD score in the distal portion of our previously defined *Gdac1* locus on mouse Chr. 2, we have objectively eliminated close to 50% of genes [8]. The major constraints for screening through the remaining 128 annotated protein-encoding and 3 miRNA genes were the high number of SNPs between the B6 and 129 strains and the multiple pathways involved in colitis [3]. Consequently, *in silico* methods were only moderately useful. An immediate rejection of 20 genes was based on instances where there was no expression in the colon and/or the pathways the genes acted in were unlikely to impact IBD. This was sometimes inferred from human mutations. We were able to use microarray data from de Buhr *et al.* to directly exclude a few genes due to lack of expression in mouse colon (de Buhr’s Supplemental Gene Expression Omnibus (GEO) data) [32]. Relying on web tools to cull more genes involved some uncertainty because the mouse SNP databases are not yet complete and *in silico* tools, such as PolyPhen, are still being refined. However, altogether we were able to discount 22 more genes due to an absence of nsSNPs and doubtful e-QTL status, or the nsSNPs present rated as benign and no differences in expression levels between strains.

After reviewing the literature on the remaining 86 genes, we selected 25 as the best colitis gene candidates based on gene function in the colon and/or pathology observed in DKO mice (i.e. apoptosis, proliferation, mucin depletion, colitis and dysbiosis). Using individual e-QTL analysis, we eliminated 9 genes because they had no nsSNPs and no evidence of cis-e-QTL status. We also downgraded the importance of 6 more genes because they had no disease-associated nsSNPs and no expression differences.

Four of the 10 remaining candidates were involved in cell cycle checkpoint/DNA repair pathways. *Bub1b*_11_ and *Casc5*_24_ could affect the pathology of *Gpx*1/2-DKO mice via DNA damage check-point regulation and apoptosis. Because their products interact, it is possible that the nsSNPs of either gene could have a significant impact on this pathway [51]. *Oip5*_40_, which functions in the cell-cycle regulation pathway, has damaging nsSNPs [52]. *Trp53bp1*_78_, which affects DNA damage responses and contributes to immune regulation, might be eliminated upon the completion of the database for B6 and 129 nsSNPs [53], [54]. Because these four genes did not have a direct interaction with GPX they were not prioritized as top candidates, but they remain of interest for future investigation as are the pathways they represent.

Three other candidates may be linked to IBD for various reasons and all have significant nsSNPs. The solute carrier, *Slc28a2*_108_, is involved in the control of extracellular adenosine pools, which, in turn, are involved in inflammation pathways. The lower expression level of *Slc28a2*_108_ we observed in the colons of 129-*Gdac1* ^129/129^ DKO mice was consistent with an expected anti-inflammatory reaction to maintain high extracellular levels of adenosine; however the levels in the 129-*Gdac1^129/129^* mice were not statistically less than the other groups of mice [55]. *Plcb2*_14_ produces a phospholipase C, which has definitive roles in inflammation [56]. *Pla2g4e*_54_ is a cis-e-QTL with one disease-associated nsSNP. However, because the PLA2G4E protein does not have PLA2 activity to produce arachidonic acid, we did not choose *Pla2g4e* as a top candidate [38]. Although the *Slc28a2*, *Plcb2* and *Pla2g4e* genes were good candidates, they were not prioritized as top candidates because their protein products do not interact with GPX directly.

*Chac1*_36_ has been linked to ER stress downstream of Chop and to an apoptosis-promoting pathway [42]. *Chac1* responds to oxidized 1-palmitoyl-2-arachidonyl-*sn*-3-glycerophosphorylcholine treatment of aortic endothelial cells [57]. This links *Chac1* to oxidative stress, although not directly to GPX1 or GPX2 [58]. The up-regulation of *Chac1* in 129-DKO mice was consistent with the observed elevated apoptosis but no ER stress was detected in 129 *Gpx*1/2-DKO mice [8]. Therefore, the cause of the significant 4–7 fold up-regulation in the colon may be linked to other pathways down-stream of oxidative stress. Also, it is unclear in which cell types *Chac1* is expressed and induced. Lusis *et al.* showed that *Chac1* induction by oxidized phospholipid occurred in HAEC (human aortic endothelial cells) but not HEK (human embryonic kidney) or HeLa (human cervical cancer) cells [42]. There was a non-significant, but noticeable difference in *Chac1* expression levels between the parental strains but the difference was significant between the *Gdac1^B6/B6^* DKO set and the *Gdac1^129/129^* DKO set. Although *Chac1* may not be a true cis-e-QTL, its synergy with the pathology may be relevant in candidate assessment. Currently, the nsSNPs status of *Chac1* is undetermined due to discrepancies in the databases. The *Chac1* and *Casc5* genes are located in two haplotype blocks shared by 129 and C3H. Their candidacy does support the assumption that *Gdac1* and *Cdcs3* are based on the same polymorphism. The uncertainties in *Chac1* nsSNP and e-QTL status prevented us from rating it as a top candidate.

The best colitis gene candidates were *Pla2g4f*_56_ and *Duox2*_103_, because their products can interact with GPXs directly and have been implicated in immune responses and colitis [37], [38], [59]. The *Pla2g4* cluster at the distal Chr. 2 produces an obscure set of intracellular enzyme activities in contrast to the well characterized *Pla2g4a* and *Pla2g4c* genes on Chr. 1 and 7, respectively [60], [61], [62]. *Pla2g4f* is the only gene in the *Pla2g4* cluster that is a candidate equal to *Duox2*. By virtue of its colon expression, possible e-QTL status, 3 significant nsSNPs, PLA2 activity and an interaction with GPX [38], [41], we considered it to be a top candidate. The arachidonic acid generated by PLA2 is essential for activation of phagocyte NADPH oxidase that is required for microbicidal activity [63]. Pla2gIVF (encoded by *Pla2g4f*) mobilizes to membrane ruffles, and possibly contributes to intestinal epithelial restitution [40]. The nsSNPs rated as damaging/disruptive by PolyPhen-2 for *Pla2g4f* and *Duox2* were shared between 129 and C3H with B6 as the outlier. Although the genes were not on shared haplotype blocks, candidacy based on nsSNPs supported the assumption of replication of *Gdac1* and *Cdcs3.*

Of the disease phenotypes used in this study, *E. coli* overgrowth was not linked to intense inflammation. *E. coli* overgrowth occurred with roughly 85% penetrance in the cecum of the 129 *Gpx*1/2-DKO mice, whereas *Gdac1^B6/B6^* reduced the penetrance to 37%. The tendency for overgrowth was shared by 129 IL10-KO mice, and others have shown the overgrowth phenotype to be a property of WT 129 rather than B6 [64], [65]. Gulati *et al*. suggest that variation in Paneth cell numbers and antimicrobial products may mediate this tendency [65]. *Gdac1* does not have genes encoding antimicrobial peptides. However, the DUOXs have antimicrobial activities in several eukaryotic systems including *Caenorhabditis elegans*, *Drosophila*, *Anopheles*, zebra fish intestine and mammalian lung [66], [67], [68], [69], [70]. NOD2 is encoded by the *IBD1* gene and interacts with DUOX2, which functions as a NOD2 effecter [71]. Furthermore, we have recently characterized lactoperoxidase (LPO) expression in colon epithelium [72]. LPO and DUOX may form a potent antimicrobial defense team in the colon to fend off microbial invasion as they do in other tissues [37], [70]. Therefore, we have selected *Duox2* and *Pla2g4f* as prime candidates for further analysis of their roles in murine colitis models and human IBD.

## Materials and Methods

### Mice

The original *Gpx*1/2-DKO colony was a mixed line of the B6 and 129 strains [8]. This line was backcrossed to B6 for 7 generations, and to 129 to produce N5, N7 and N10 cohorts [8]. All data for *Gdac1* genetics were obtained from mice fed semi-purified diets (Harland Teklad; casein, sucrose, corn oil; TD 06306 and TD 06307), designed to mimic LabDiets 5020 (10% corn oil, Purina) and 5001 (5% corn oil) for calories and macronutrients and using AIN76A vitamin and micronutrient specifications [15]. These diets reduced disease severity relative to LabDiet formulations [8], [15]. To produce homozygous DKO offspring efficiently, it was necessary to use semi-purified diets to prevent high mortality of homozygous DKO male breeders before reaching 35 days of age when on LabDiets. Studies reported here were approved by the City of Hope Institutional Animal & Use Committee.

### Refined *Gdac1* Marker Panel

A denser SNP marker panel was established concentrating on the *Gdac1* region of Chr. 2 and some of the flanking area (Table 1) [8]. Additional SNPs were examined on Chr 1: 88 and 142 mbp as well as Chr 3: 64 and 118 mbp because B6 alleles were detected by a genome-wide scan of ten 129 N7 mice performed by the Jackson Laboratory Genome Scanning Service (Bar Harbor, ME) using 141 markers to cover the 19 autosomes at ∼20 mbp interval (Table 1 and [8]). SNPs were obtained from the Jackson Laboratory MGI SNP database (www.Jax.org) and the flanking sequences were screened for repeats in RepeatMasker (www.repeatmasker.org/cgi/bin/WEBRepeatMasker). Primers were made for the MassARRAY iPLEX Gold system by Sequenom, San Diego, CA and primer sequences are available on request. All DNA samples from the 129 N7 cohort described previously were rescreened [8].

### Phenotypes Used in Mapping

Disease activity index (DAI): One hundred ninety-nine mice were analyzed for DAI, a post-hoc semi-quantitative appraisal of mouse health from 8 to 22 days of age or presentation of morbidity. DAI criteria (listed in Table 2) were modified from traditional criteria. Our criteria accounted for growth arrest and did not account for blood in the stool [73]. The final score was based on adding the findings from the growth/wasting column and the diarrhea column, so that the scores ranged from 0 to 8.

Colon length and pathology: Colon length was measured on 117 mice, and colon pathology scores (based on H&E stained sections) were appraised on 92 mice [8]. Pathology scores of 0–6 generally reflect presence of crypt apoptosis/hyperproliferation and mucin depletion without overt signs of inflammation. When the score is above 6, acute inflammation is generally evident with neutrophil infiltration, gland abscesses or erosion of the epithelium [8], [15].

*E. coli* overgrowth: The cecum contents were analyzed for *E. coli* colony forming units (CFU)/gm on LB plates grown aerobically at 37°C for 18–22 hours. The cecum is a disease site in these mice [8], [15]. Large colonies were scored as *E. coli*, and less frequently detected small colonies were identified as *Enterococcus sp.* (*hirae, gallinarum* or *faecalis*). The colony identity was established from the sequence of rDNA amplified from single colonies for both *E. coli* and *Enterococcus sp.* and *E. coli* colonies also were verified by the Clinical Microbiology Laboratory at COH [15]. Spot checks were performed on randomly selected large colonies throughout the project to confirm their identities. Single dilutions of cecal contents were plated with an approximate sensitivity of 2×10^6^–1×10^7^ CFU/gm [8], [15]. Zero colonies were entered as a default of 1x10^6^ CFU/gm for statistical analysis in R/QTL, which was empirically determined to be the upper limit for healthy mice at this age [15], [74]. Log10 transformed CFU/gm was used as the phenotype parameter. One hundred seventeen samples were used for R/QTL analysis.

### R/QTL Interval Mapping Analysis

Statistical associations of markers and phenotypes were performed to identify the loci underlying the traits. Interval mapping was performed with the R/QTL interface, J/QTL (version 1.3.1; cgd.jax.org). The LOD thresholds were calculated using 2000 permutations. The marker physical locations were converted to genetic locations using the Mouse Map Converter (cgd.jax.org/tools/tools.shtml). The genetic length was increased to adjust for the multiple generations. Log of the odds (LOD) scores and the 95% confidence interval were established by the program (Bayesian credible interval) [75]. Our original marker spacing across Chr. 2 was at 10 cM intervals [8]. Here, we decreased the marker intervals to 2.5–3.5 cM in the core of the *Gdac1* region to detect recombination internal to the original markers and obtain an estimate of the location (Table 1). The QTLs were positioned by the interval mapping program that calculates maximum likelihood estimates (LOD scores) at and between markers using quantitative phenotype data. The scores are a measure of the strength of association of a trait and genotype stated as the log_10_ of the likelihood of the odds ratio (LOD). LOD scores of 3.3 and 4.3 or greater are generally considered statistically significant evidence of association in backcrosses and intercrosses involving one generation, respectively [76]. In this case, the thresholds for the colon pathology, DAI and CFU phenotypes (6.8, 15.5 and 8.4; α Threshold = 0.05) were higher as a consequence of the adjustment for multiple generations.

### RT-PCR for Evaluation of Genes as e-QTLs

Mice were euthanized by CO_2_ asphyxiation. Distal colon tissues were dissected out and stored in RNAlater (Qiagen). For the synthesis of cDNA, colon tissues were homogenized with a Polytron homogenizer (PT 1200E: Brinkmann Kinematica, Fisher Scientific) and sonicated. Total RNA was isolated using the RNeasy Mini kit (Qiagen). cDNA was synthesized from 2 µg of total RNA using M-MLV reverse transcriptase (Promega, Madison, WI, USA) in the presence of 1 µg of random hexamers (Invitrogen). Real-time quantitative PCR (qPCR) was performed with the Eva qPCR SuperMix kit containing SYBR green dye (Biochain Institute, Hayward, CA, USA) using the iQ5 Detection system (Bio-Rad Laboratories, Hercules, CA, USA).

Data was analyzed with Bio-Rad iQ5 Optical System Standard Edition, version 2.0 software. The primer sequences for 25 genes are listed in Table 3. Briefly, standard curves for each primer set were generated from a serial dilution of pooled test samples plotted with x-axis of log starting quantity (SQ) and y-axis of threshold cycle (Ct). Only those results obtained with PCR efficiency between 80–120% and correlation coefficient (R^2^) between 0.95–1.00 (obtained from the standard curve) were used. The cDNA quantity of each sample was determined from the Ct value based on the standard curve, and then normalized to β-actin or 36B4 for acidic ribosomal phosphoprotein P0 [77], both showed similar results. Each assay was performed in duplicate. For screening purposes, P≤0.1 was considered of interest. The relevant comparisons in this evaluation were between the parental strains and between 129-*Gdac1^B6/B6^* and 129-*Gdac1^129/129^* mice. Finding a cis-e-QTL requires a significant difference in both sets. 129-*Gdac1* mouse designation as *B6/B6* and *129/129* was based on genotyping with marker SNPs at 118.8 and 122.1 mbp.

### In Silico Analysis

The distal Chr. 2 gene list was obtained from the National Center for Biotechnology Information (NCBI). It was updated for revisions in gene nomenclature, addition of new open-reading frames and annotation of genes in PubMed, MGD (Mouse genome database), NCBI and Ensembl (www.ensembl.org) [78]. Annotated genes were evaluated for involvement in pathways that were relevant to the pathology of *Gpx*1/2-DKO mice by literature searches and reports of KOs and/or use of agents such as dextran sodium sulfate (DSS), when available, in addition to mutations in human genes. PolyPhen-2 was used to evaluate nsSNPs for potential significant impact on protein function [33]. One GEO dataset, available as supplemental data for de Buhr *et al*., had colon microarray expression data for approximately one-third of the genes in the *Gdac1/Cdcs3* loci from the B6 and C3H strains (NCBI: GEO; GSM39288–GSM39297) [32]. This analysis set the threshold for biological effects at a 1.5x expression level difference between strains. A manual survey of the results suggested that statistical significance was unlikely at less than 1.5x difference. These results were combined with available literature to determine if gene expression was significant in the colon, if the mouse strain background could have a significant impact on levels, and to evaluate selected RT-PCR results performed for this study.

## Supporting Information

Figure S1 **LOD plot for the colon length.** See legend to Figure 2.
(TIF)

Figure S2 **LOD plot for disease activity index.**
(TIF)

Figure S3 **LOD plot for log10 E. coli/gm cecal contents.**
(TIF)

Figure S4 **Verification of R-QTL result.** Panel A shows genotypes of 4 groups of mice manually analyzed for R-QTL verification. The number in the arrow is the mbp of SNP markers used for genotyping. B6/B6 and 129/129 genotypes are shown in solid black and white boxes, respectively. The shaded gray box indicates either B6/B6, 129/129 or B6/129 genotypes present in those regions in individual mice. A *Gdac1* congenic line established in the 129 strain mice. The differential segment of the mice is anchored at 118.8 mbp at the proximal end and the distal end is at 137 mbp. The group of N7 B6/B6-122 B6/B6 distal consists of mice typed as B6/B6 across the 118.8–132 mbp interval. The N7 129/129-122 B6/B6 distal group consists of mice that typed 129/129 at 118.8 and 122.1 mbp and B6/B6 at 127 and 132 mbp. The 129 N10 mice are 129/129 throughout. Panels B–E show the same phenotypes as in Figure 1. Letters indicate significant differences in means for panels C and E or medians for panels B and D; where a>b; P≤0.05; 1-way ANOVA.
(PPT)

Figure S5 **Detailed haplotype block analysis on the B6, 129 and C3H strains across** ***Gdac1*** **.** The comparison of C3H and 129 is shown as diamonds, where a value of zero represents identical haplotypes and y-axis shows the values representing a metric of dissimilarity of haplotypes for each pair of strains. In the region from 117.7 to 124 mbp, the C3H and 129 haplotypes had greater resemblance to each other than to B6 (129 vs. B6: black squares; C3H vs. B6: open triangles). The location of the *Pla2g4f* (proximal) and *Duox2* (distal) genes are indicated by the small horizontal red bars. Data were retrieved from CGD (http://msub.csbio.unc.edu/) and MGD at the MGI website (04/2012).
(PPT)

Table S1 ***Gdac1*** **gene list and analysis of candidacy.** The 128 gene list excludes 30 entries that include pseudogenes, tRNA genes and predicted but un-annotated open reading frames. Two validated and one putative miRNA are listed as #a where # is the nearest proximal gene. The shaded entries are genes that were eliminated in the preliminary analysis. Each column is defined below: 1. Number of genes. The subscript numbers following the gene symbols in the text refer to the entry number in the Table. 2. Gene symbols are in the order of proximal to distal (116.9–125.6 mbp) in the physical map. 3. Gene names were obtained from NCBI. 4. nsSNPs were mined from MGD and CGD. nsSNPs are either present (Y), absent (N; means no for B6 vs. 129), maybe (means data is missing for B6 and/or 129, but at least one inbred strain from the full list has nsSNPs at a given position) or problem with IDs (nsSNPs listed at multiple locations or other problems with database listing). 5. Evaluation of nsSNPs (SNP eval) with PolyPhen-2 program. “Benign” means that the amino acid variations are unlikely to alter protein functionality or structure. “Damaging SNP” means that variant amino acids are substantially different and therefore, are likely to have an impact on the protein function. “Uneval” means unevaluated; the nsSNP and corresponding amino acids conflict with amino acid sequence downloaded from MGD, so cannot be analyzed. PolyPhen-2 nsSNP evaluations are not definitive. Therefore, genes with benign nsSNPs and no expression differences cannot be positively eliminated as candidates. 6. qRT-PCR evaluation of relative mRNA levels of 25 selected genes in 6 WT B6 colons after normalization with β-actin. 7. qRT-PCR evaluation of relative mRNA levels of 25 selected genes in 6 WT 129 colons after normalization with β-actin. 8. qRT-PCR evaluation of relative mRNA levels of 25 selected genes in 6 DKO 129-Gdac1^B6/B6^ colons after normalization with β-actin. 9. qRT-PCR evaluation of relative mRNA levels of 25 selected genes in 6 DKO 129-Gdac1^129/129^ colons after normalization with β-actin. 10. P shows the p-value for the *Gdac1* comparison (columns 8 and 9). 11. e-QTL. N means no variation in levels found between parental strains or *Gdac1* variants. Maybe means difference found between *Gdac1* variants, however, in all but the *Pla2g4e* gene, the differences are likely the result of pathology or inflammation. Y means cis-e-QTL based on both parental strain and *Gdac1* variants. A cis-e-QTL is the case where the difference observed between the parental strains is preserved between 129-*Gdac1^129/129^* DKO and 129-*Gdac1 ^B6/B6^.* 12. e-QTL. Clarification of the e-QTL difference based on groupings; for example, WT vs. DKO. A major finding was an outlier 129-*Gdac1^129/129^* DKO expression level, suggesting a reaction to pathology or inflammation. 13. Pathway analysis or expression levels obtained through *in silico* analysis. “Unlikely” means that either the gene was not expressed in the colon and/or the pathways involved are unlikely to impact colitis. 14. Determination of candidate genes. D is discounted for the present. Undecided is no expression data, and/or SNPs can’t be evaluated, or expression passed 1.5x threshold in de Buhr *et al.* analysis. Y is a candidate gene. N is not a candidate. The order of candidacy is Y>D = Undecided>N. 15. Column 14. Summary of the reason for candidacy. A candidate gene has either potentially disruptive amino acid variants or e-QTL status, or both. No entry means that there was insufficient information for evaluation at this time or that the gene was among the 25 selected for further analysis and is discussed in the text.
(XLS)
