# Supplementary figures and images for: Analysis of Candidate Colitis Genes in the Gdac1 Locus of Mice Deficient in Glutathione Peroxidase-1 and -2
Source: PLoS One. 2012 Sep 6;7(9):e44262. doi: 10.1371/journal.pone.0044262 (PMC3435402; doi:10.1371/journal.pone.0044262)

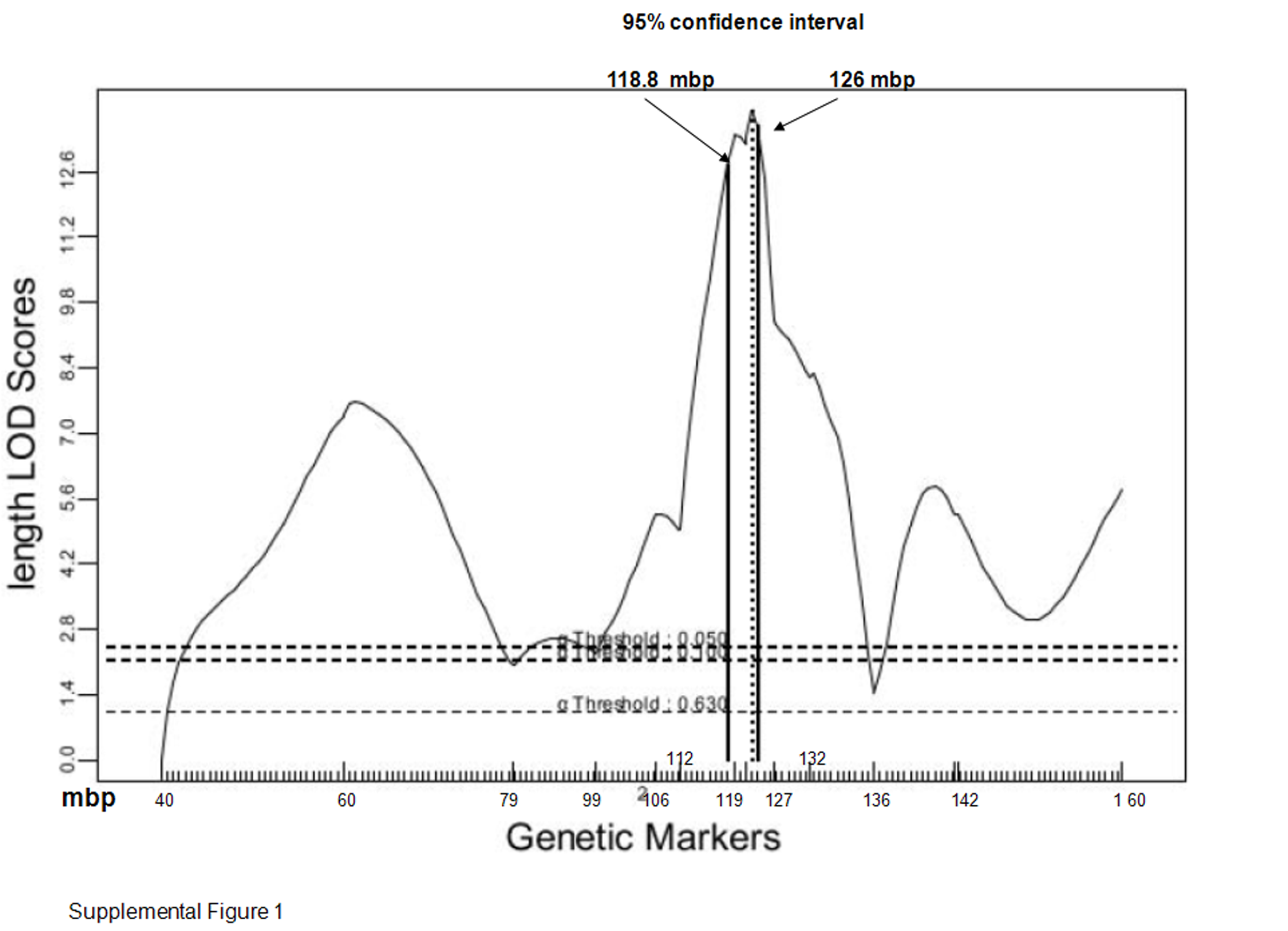

Supplement: Figure S1 — LOD plot for the colon length. See legend to Figure 2. (TIF) [file pone.0044262.s001.tif]

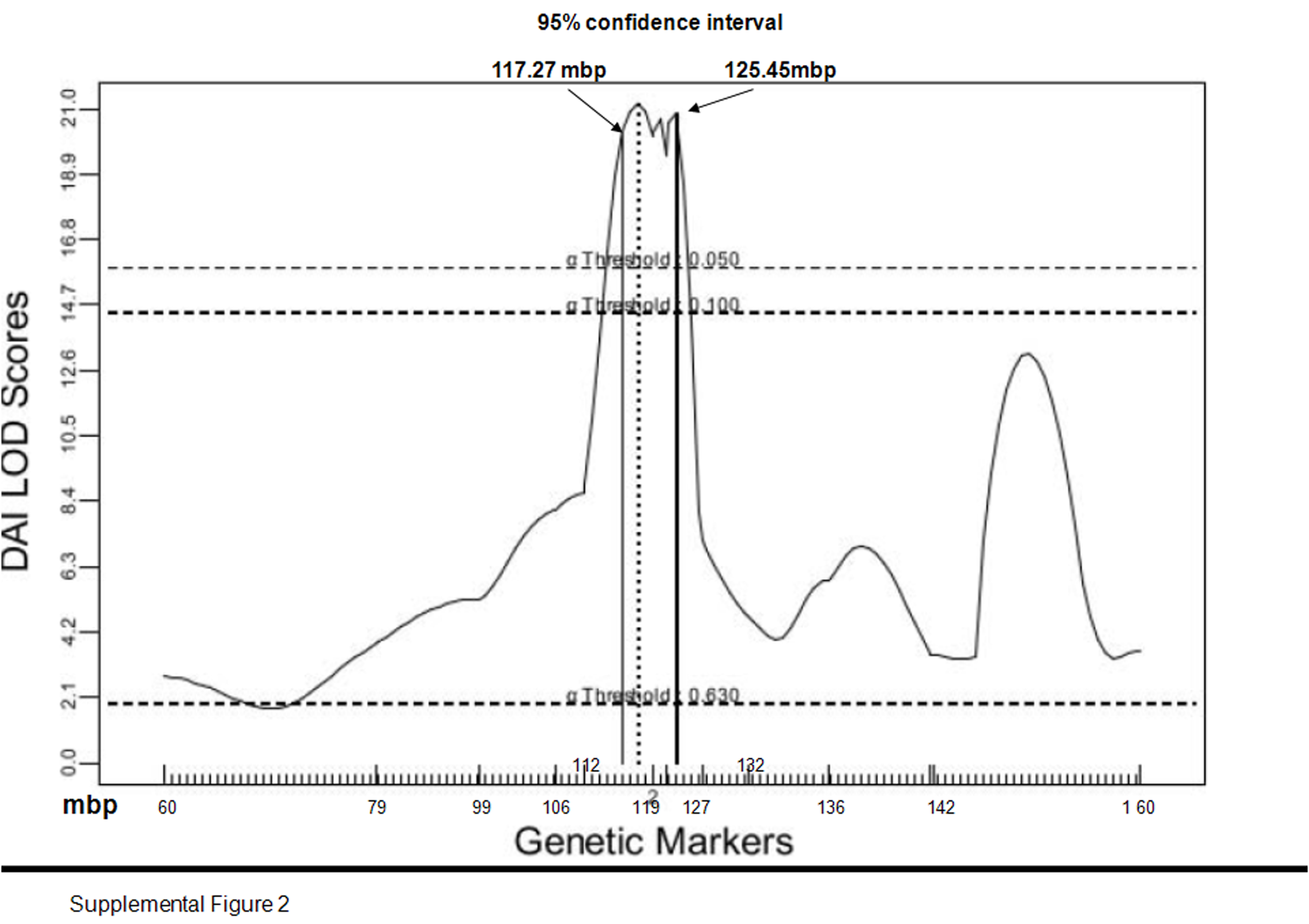

Supplement: Figure S2 — LOD plot for disease activity index. (TIF) [file pone.0044262.s002.tif]

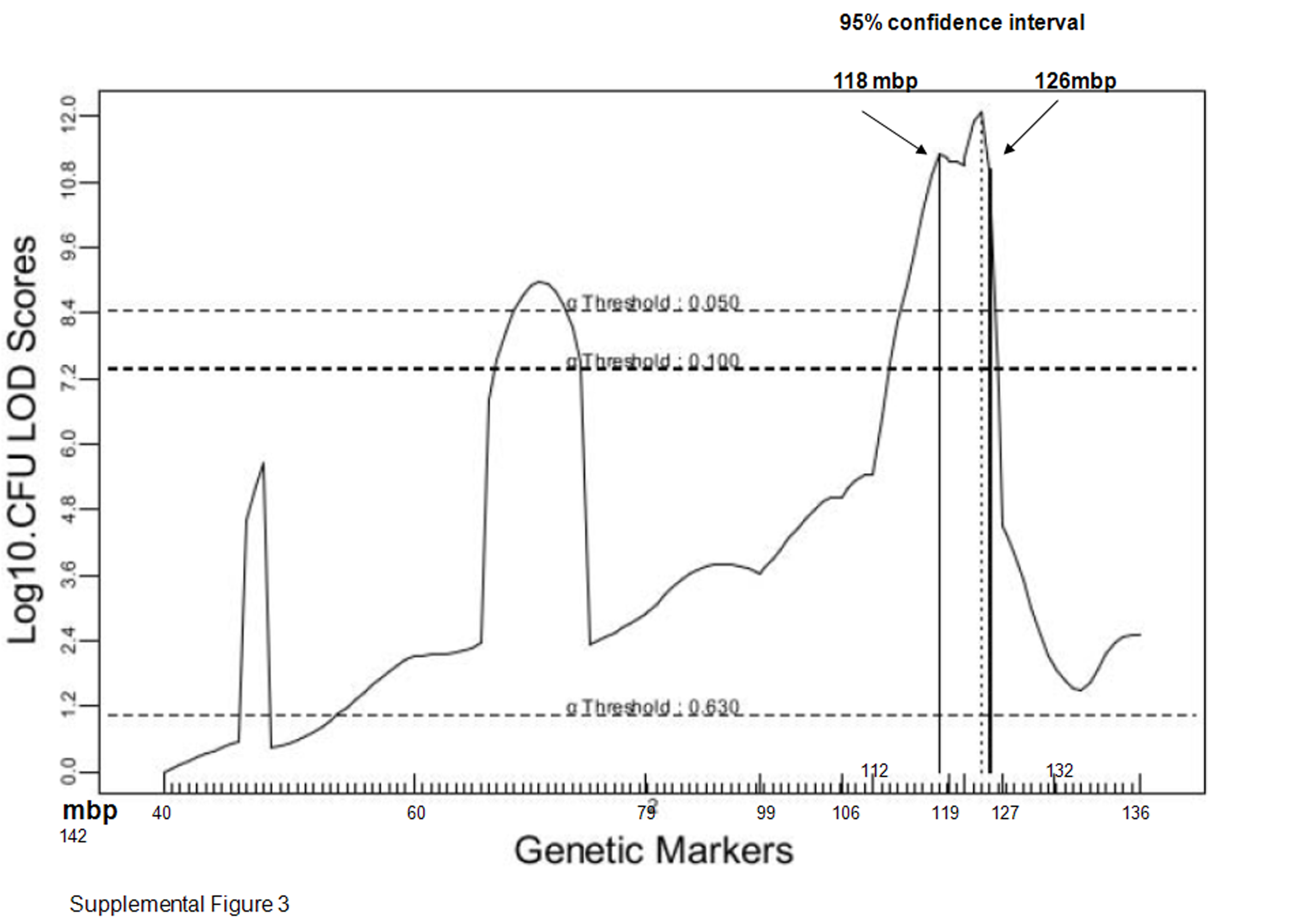

Supplement: Figure S3 — LOD plot for log10 E. coli/gm cecal contents. (TIF) [file pone.0044262.s003.tif]

## Slide 1
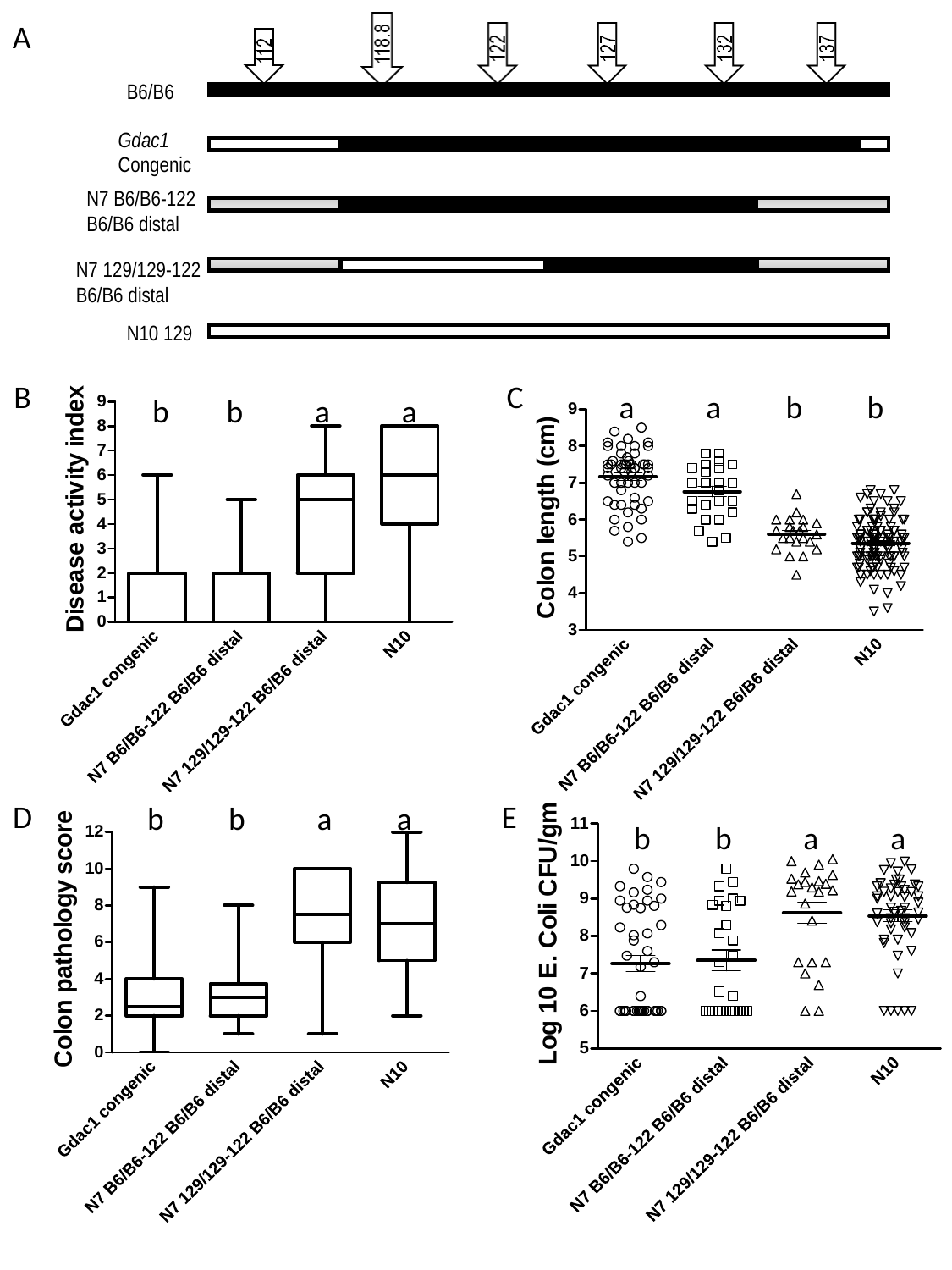

A
B6/B6
Gdac1 Congenic
N7 129/129-122 B6/B6 distal
N10 129
N7 B6/B6-122
B6/B6 distal
B
C
a a b b
 b b a a
D
E
 b b a a
 b b a a

Supplement: Figure S4 — Verification of R-QTL result. Panel A shows genotypes of 4 groups of mice manually analyzed for R-QTL verification. The number in the arrow is the mbp of SNP markers used for genotyping. B6/B6 and 129/129 genotypes are shown in solid black and white boxes, respectively. The shaded gray box indicates either B6/B6, 129/129 or B6/129 genotypes present in those regions in individual mice. A Gdac1 congenic line established in the 129 strain mice. The differential segment of the mice is anchored at 118.8 mbp at the proximal end and the distal end is at 137 mbp. The group of N7 B6/B6-122 B6/B6 distal consists of mice typed as B6/B6 across the 118.8–132 mbp interval. The N7 129/129-122 B6/B6 distal group consists of mice that typed 129/129 at 118.8 and 122.1 mbp and B6/B6 at 127 and 132 mbp. The 129 N10 mice are 129/129 throughout. Panels B–E show the same phenotypes as in Figure 1. Letters indicate significant differences in means for panels C and E or medians for panels B and D; where a>b; P≤0.05; 1-way ANOVA. (PPT) [file pone.0044262.s004.ppt]

## Slide 1
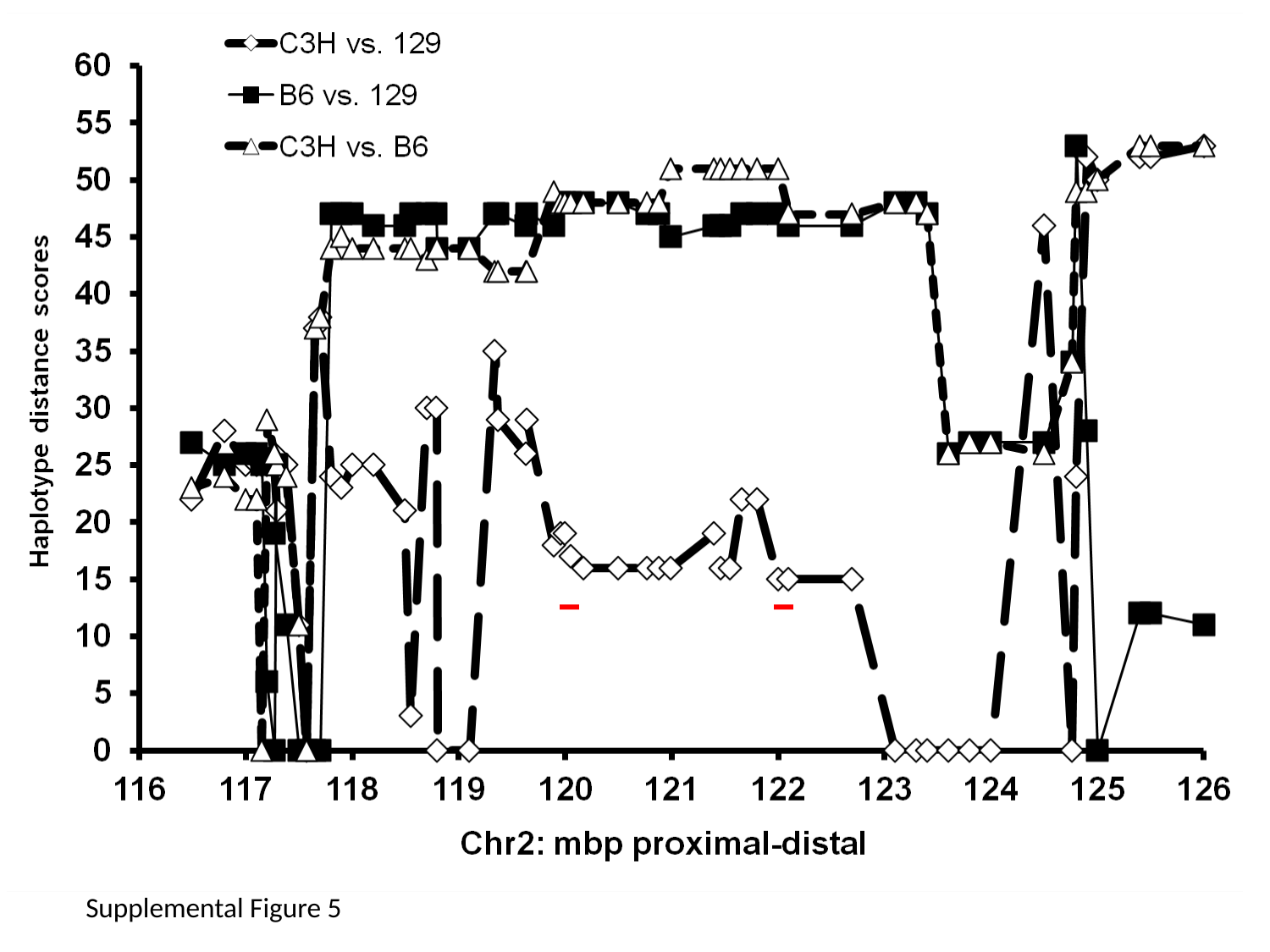

Supplemental Figure 5

Supplement: Figure S5 — Detailed haplotype block analysis on the B6, 129 and C3H strains across Gdac1 . The comparison of C3H and 129 is shown as diamonds, where a value of zero represents identical haplotypes and y-axis shows the values representing a metric of dissimilarity of haplotypes for each pair of strains. In the region from 117.7 to 124 mbp, the C3H and 129 haplotypes had greater resemblance to each other than to B6 (129 vs. B6: black squares; C3H vs. B6: open triangles). The location of the Pla2g4f (proximal) and Duox2 (distal) genes are indicated by the small horizontal red bars. Data were retrieved from CGD (http://msub.csbio.unc.edu/) and MGD at the MGI website (04/2012). (PPT) [file pone.0044262.s005.ppt]
